# Correlation between a New Point-Shear Wave Elastography Device (X+pSWE) with Liver Histology and 2D-SWE (SSI) for Liver Stiffness Quantification in Chronic Liver Disease

**DOI:** 10.3390/diagnostics13101743

**Published:** 2023-05-15

**Authors:** Matteo Garcovich, Mattia Paratore, Laura Riccardi, Maria Assunta Zocco, Maria Elena Ainora, Geltrude Mingrone, Antonio Gasbarrini, Maurizio Pompili

**Affiliations:** 1 Medicina Interna e Gastroenterologia, CEMAD Digestive Disease Center, Fondazione Policlinico Universitario A. Gemelli IRCCS, Largo A. Gemelli 8, 00168 Rome, Italy; 2 Dipartimento di Medicina e Chirurgia Traslazionale, Università Cattolica del Sacro Cuore, 00168 Rome, Italy; 3 U.O.C. Patologie dell’Obesità, Fondazione Policlinico Universitario Agostino Gemelli IRCCS, 00168 Rome, Italy; 4 Internal Medicine and Gastroenterology-Hepatology Unit, Fondazione Policlinico Universitario Agostino Gemelli IRCCS, 00168 Rome, Italy; 5 Translational Medicine and Surgery Department, Università Cattolica del Sacro Cuore, 00168 Rome, Italy

**Keywords:** shear-wave elastography, liver fibrosis, liver stiffness, Alpinion, liver biopsy

## Abstract

Background: The aim of this study was to investigate the feasibility, the correlation with previously validated 2D-SWE by supersonic imagine (SSI), and the accuracy in fibrosis-staging of a novel point shear-wave elastography device (X+pSWE) in patients with chronic liver disease. Methods: This prospective study included 253 patients with chronic liver diseases, without comorbidities potentially affecting liver stiffness. All patients underwent X+pSWE and 2D-SWE with SSI. Among them 122 patients also underwent liver biopsy and were classified according to histologic fibrosis. Agreement between the equipment was assessed with Pearson coefficient and Bland–Altman analysis, while receiver operator characteristic curve (ROC) analysis with Youden index was used to establish thresholds for fibrosis staging. Results: A very good correlation was found between X+pSWE and 2D-SWE with SSI (r2 = 0.94; *p* < 0.001), with X+pSWE average liver stiffness values 0.24 kPa lower than those obtained with SSI. AUROC of X+pSWE for the staging of significant fibrosis (F2), severe fibrosis (F3) and cirrhosis (F4) using SSI as a reference standard was 0.96 (95% CI, 0.93–0.99), 0.98 (95% CI, 0.97–1) and 0.99 (95% CI, 0.98–1), respectively. The best cut-off values for diagnosing fibrosis ≥F2, ≥F3 and F4 were, respectively, 6.9, 8.5 and 12 for X+pSWE. According to histologic classification, X+pSWE correctly identified 93 out of 113 patients (82%) for F ≥ 2 and 101 out of 113 patients (89%) for F ≥ 3 using the aforementioned cut-off values. Conclusion: X+pSWE is a useful novel non-invasive technique for staging liver fibrosis in patients with chronic liver disease.

## 1. Introduction

Liver fibrosis is a pathological process characterized by increased production and deposition of collagen into the hepatic parenchyma [1] by activated stellate cells in response to a variety of noxious and inflammatory stimuli in chronic liver disease (CLD) [2]. Chronic hepatitis B (HBV) and C (HCV), alcohol-use disorder hepatopathy and non-alcoholic fatty liver disease (NAFLD) are the most frequent causes of hepatic injury and consequently of liver fibrosis [3,4,5]. Moreover, liver fibrosis is a dynamic and progressive process that can lead to cirrhosis and its complications, one of the leading causes of disability and one of the diseases with the highest cost burden on the global health-care system [6]. Therefore, it is essential to identify patients with CLD at an early stage of liver fibrosis and successfully treat them prior to the development of cirrhosis.

Although hepatic biopsy (with its well-known limitations) is still considered the gold standard for liver fibrosis evaluation, shear-wave elastography (SWE) techniques have largely replaced liver biopsies in routine clinical practice by measuring liver stiffness (LS), which is a non-invasive surrogate of liver fibrosis. Transient elastography (TE) is one of the first non-invasive techniques utilized for this aim and is considered the most “experienced” non-invasive test for the evaluation of LS in viral CLD, having a high negative predictive value for the detection of cirrhosis. The major disadvantage of the TE is that it cannot be easily utilized in obese or ascitic individuals [7,8,9]. In contrast, magnetic resonance elastography (MRE) is a technique that enables a thorough assessment of liver parenchyma, with great diagnostic accuracy and outstanding capacity to differentiate the various degrees of liver fibrosis. Due to high cost and time restrictions, it is not widespread for routine applications in many countries [10]. For the non-invasive investigation of LS, multiple applications of ultrasound (US)-based SWE, such as point-SWE (p-SWE) or 2D-SWE implemented on several types of sonographic devices, have been developed in recent years. Among them, 2D-SWE installed in supersonic imagine (SSI) machines is one of the most validated techniques [9,11]. Unfortunately, due to intersystem variability, the lack of universal cutoffs is the most significant limitation for many SWE devices. Therefore, the European Federation of Societies for Ultrasound in Medicine and Biology (EFSUMB) recommends the adoption of system-specific cut-off values established by comparing studies with the reference techniques for LS stratification [9,11].

The X-Cube 90 platform is one of the most recent devices. Introduced in 2021 by Alpinion (Seoul, Republic of Korea), it is embedded with a point-SWE (X+pSWE). Due to its recent release, no study has, to the best of our knowledge, been published on diagnostic accuracy and technical performance of this new SWE technique. The aim of this study was therefore to assess for the first time the correlation between X+pSWE and other validated SWE techniques and liver biopsy for non-invasive evaluation of liver fibrosis in patients with different etiologies of CLD. The secondary aims of the study were to assess technical feasibility of this new technology and, for the first time, to establish specific LS thresholds for fibrosis staging using as a reference standard both SSI and liver biopsy [12].

## 2. Materials and Methods

### 2.1. Study Design and Patients

In this prospective single-center cross-sectional study, consecutive patients with CLD referred to our US Unit for LS measurement were enrolled between February 2022 and October 2022. CLD was characterized as abnormal liver-function tests (serum transaminases and/or gamma-glutamyl transferase or alkaline phosphatase) persisting for more than six months, with or without US evidence of chronic illness (bright liver or findings of advanced hepatic disease). Based on the indication of the attending physician, a subgroup of patients underwent percutaneous liver biopsy in order to identify and stage CLD.

Subjects were required to fast for a minimum of 6 h. The exclusion criteria were age <18 years, decompensated liver cirrhosis, severe extrahepatic comorbidities (in particular cardiac and respiratory), liver malignancies, and highly elevated LS values (>50 kilopascal, kPa) and liver function tests (>5× ULN of alanine/aspartate transaminases). For each patient demographic (age, sex) and clinical information (i.e., liver disease etiology, history of decompensated CLD and body mass index (BMI)) findings were recorded. Standard laboratory tests and liver-function tests no older than one month together with customary US findings (such as the presence of ascites and US grading of hepatic steatosis) were also collected.

### 2.2. Ultrasound Shear-Wave Elastography

**Figure 1:**
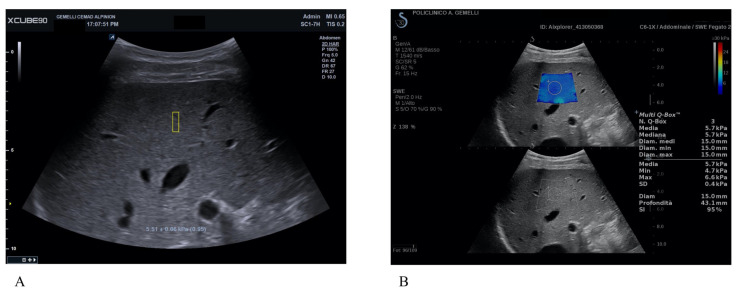
Shear-wave elastography (SWE) in a patient with no clinically significant liver fibrosis (F0-1). (**A**) X+pSWE with a median value of 5.5 kPa after 10 valid measurements; the number in the brackets (0.95) represents a reliability indicator, which provides immediate feedback about measurement quality for each single measurement specifically developed by Alpinion. (**B**) SSI with a median value of 5.7 kPa after 3 measurements. In 2D-SWE techniques, US images show the stiffness color map, with a homogenous blue filling that correspond to low elasticity values on the color scale.

All enrolled patients underwent non-invasive measurement of LS with X-CUBE 90 (X+pSWE) (Alpinion Medical Systems Co., Ltd., Seoul, Republic of Korea) using the convex probe SC1-7H and with real-time 2D-SWE with Mach 30 Aixplorer (SSI) (Aix-en-Provence, France) using the convex probe XC6-1. The exam was performed by two expert operators with more than five years of experience with elastography techniques. The patients were assessed while supine, with the right arm maximally abducted. The probe was positioned in an intercostal region above the liver’s right lobe. During the evaluation, patients were instructed to maintain a semi breath hold, avoiding both the Valsalva maneuver and deep inspiration [9]. Ten measurements for each patient with X+pSWE were made, registering median values in kPa (M), interquartile range (IQR) and the ratio between IQR and M (IQR/M) (Figure 1A). As a further quality indicator, X+pSWE provided a number (0 to 1.00) after every LS measurement. As per vendor-specific recommendations, only acquisitions with values >0.80 were deemed reliable. For 2D-SWE with SSI, five measurements were performed using the same technique in a region of interest (ROI) of 10–15 mm in a central area of the colorimetric map showing the best signal homogeneity, registering M in kPa and stability index (SI) (Figure 1B).

### 2.3. Ultrasound-Guided Liver Biopsy

A subgroup of patients underwent liver biopsy on the same day that elastography recordings were performed. Liver biopsy was performed under local anesthesia with an ultrasound-guided approach with a 17- or 18-gauge semi-automatic Menghini (Surecut, TSK Laboratory, Tochigi, Japan), taking from one to two samples. There were no major significant adverse events. Only specimens 15 mm in length and containing at least eight complete portal tracts were considered suitable for the histological analysis, which was conducted by a single liver pathologist blinded for clinical and US data. Samples were classified into five degrees of fibrosis (F0–4) according to METAVIR [13]. The protocol adhered to the ethical criteria of the local institutional research committee (Comitato Etico, Fondazione Policlinico A. Gemelli IRCCS) and the Declaration of Helsinki of 1964 and its later revisions. Each subject’s permission was acquired with knowledge of the risks involved.

### 2.4. Statistical Analysis

Statistical analysis was performed by using the Statistical Package for Social Science (SPSS 22.0; SPSS Inc., Chicago, IL, USA). Descriptive statistics were produced for demographic, anthropometric, clinical and laboratoristic findings, and expressed as fractions and percentages or as the median and the IQR. Method comparisons were evaluated using Bland–Altman analysis and Pearson coefficient. The Bland–Altman limits of agreement (LOA) and their 95% confidence interval (CI) between methods were also reported and represented the interval within which the absolute difference between two test results, even with a high agreement or concordance, were expected to lie with a probability of 95%. The area under the ROC curve (AUROC) and the 95% CI were calculated to identify F2, F3 and F4 fibrosis stages, using cut-off values proposed by Hermann for SSI as reference [13]. X+pSWE optimal stiffness thresholds for significant fibrosis, severe fibrosis and cirrhosis were identified from the highest Youden index. Sensitivity, specificity, positive predictive value, negative predictive value and likelihood ratios were then calculated. As usual, we categorized the AUROC as excellent if it was above 0.9, good if it was between 0.8 and 0.9, and fair if it was between 0.7 and 0.8. For a subgroup of patients who underwent liver biopsy as well as LS measurement with SSI and X+pSWE techniques the cut-off values identified from the previous analysis were used in order to assess their performance in the fibrosis staging as compared to liver histology. For this sub-group analysis, due to the paucity of patients with severe fibrosis (F3) and cirrhosis (F4), we stratified patients into three groups: F0–1 as “no significant fibrosis”, F ≥ 2 as “significant fibrosis” and F ≥ 3 as severe fibrosis/cirrhosis.

All reported *p*-values were two-sided. Only *p*-values ≤0.05 were considered statistically significant.

## 3. Results

### 3.1. Study Population

In our study cohort a total of 253 patients were prospectively enrolled from February 2022 to October 2022. Descriptive statistics of the patients are reported in Table 1. Among the whole cohort included in the analysis, 125 subjects were female with the more prevalent etiology being non-alcoholic fatty liver disease (NAFLD), which represented 60% of the entire population. Blood tests confirmed stability of liver disease and showed only a poor necro-inflammatory and cholestatic activity. Furthermore, none of the patients presented with ascites on US scan. It is important to highlight that a remarkable subgroup of patients had a body mass index (BMI) >25 kg/m^2^, with 33% being overweight and more than 37% belonging to the obesity status.

A subgroup of patients (n = 122) underwent liver biopsy on the same day as LS measurements, but two samples/patients were excluded from the sub-group analysis due to an inadequate liver biopsy specimen. The majority of these patients had a final diagnosis of NAFLD (n = 76), while the others showed a histological picture consistent with cholestatic/autoimmune disorders (n = 22), viral hepatitis (n = 4) or other liver diseases (n = 18). The vast majority of fibrosis conditions was scored as F0 (n = 7) or F1 (n = 78), while only 35 patients were classified as F2 or higher (F2, n = 18; F3, n = 10; F4, n = 7). The “liver biopsy” subgroup had a mean BMI similar to the entire study population (28 kg/m^2^ vs. 29 kg/m^2^).

### 3.2. SWE Feasibility

Overall technical feasibility was determined after the exclusion of unreliable technical and/or measurement failures. Based on international guideline indications, “technical failure” was defined as the inability to measure LS in a homogeneous ROI of at least 10 mm for 2D-SSI or as no successful measurement after 10 attempts for X+pSWE. “Unreliable measurements” were defined by an SI <80% and an IQR/M ratio >0.30 for SSI and X+pSWE techniques, respectively [11]. All the others were considered “technically feasible”. The higher technical feasibility was obtained by SSI, with 248/253 feasible exams (98%). Feasibility was also excellent for X+pSWE with 243/253 feasible exams (96%). In particular, technical failures and unreliable measurements occurred, respectively, in four and one patients with 2D-SSI, while ten patients were not included in the final analysis mostly because of unreliable measurements rather than technical failures of X+pSWE (eight patients vs. two patients). As compared to patients with successful LS measurements, the majority of technical failures/unreliable results for both SWE techniques occurred because of high BMI values and a skin–liver capsule distance >5 cm.

### 3.3. Concordance Analysis of X+pSWE Technique with SSI

**Figure 2:**
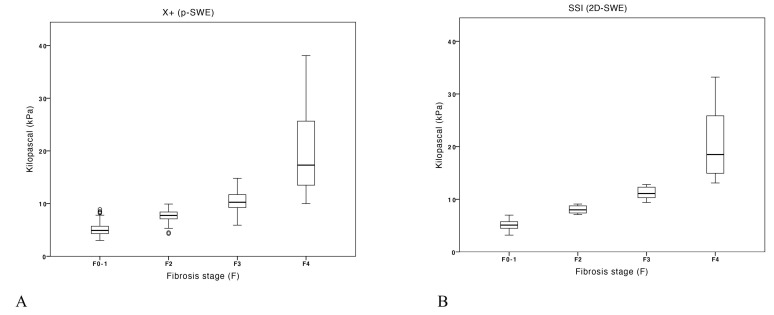
Box-plot graphic for mean liver stiffness values obtained by X+pSWE (**A**) and by SSI (**B**) techniques.

The median values of LS obtained with the two devices for the various stages of fibrosis (no–mild fibrosis (F0–F1), significant fibrosis (F2), severe fibrosis (F3) and cirrhosis (F4)) are reported in Table 2 and depicted in Figure 2. Briefly, LS measured with the X+pSWE technique showed essentially similar values as compared to SSI for early stages of liver fibrosis and slightly lower for severe fibrosis and cirrhosis.

X+pSWE showed a high correlation with fibrosis as assessed by SSI (r2 = 0.90; *p* < 0.001), and a fair correlation with platelet count (r2 = −0.33; *p* < 0.001). No significant correlation with the degree of steatosis or other study variables was found.

**Figure 3:**
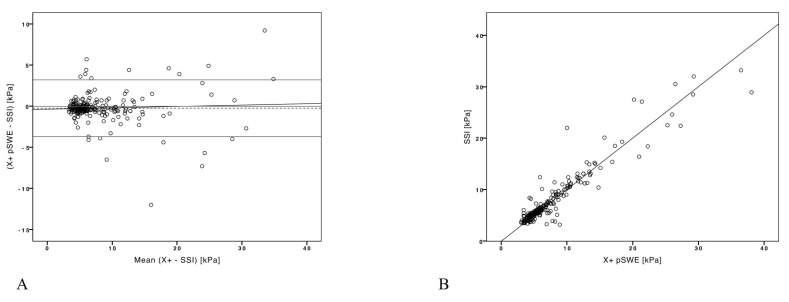
Bland–Altman plot of SSI and X+pSWE. (**A**) Bland–Altman plot shows mean difference (−0.3 kPa; dashed thick horizontal line) with respective confidence interval (CI, −2.4 to +2.9 kPa; dashed thin horizontal lines). (**B**) Scatter plot showing the correlation between SSI and X+pSWE.

Bland–Altman analysis showed that X+pSWE average values were 0.24 kPa (CI, −3.7 to +3.2 kPa) lower than those obtained with SSI, with a good correlation between the two techniques (r2 = 0.94; *p* < 0.001) (Figure 3).

### 3.4. Cut-Off Values and Performance Analysis

The AUROC analysis allowed us to identify optimal cut-off points for non-invasive LS quantification with X+pSWE, using SSI cut-off thresholds proposed by Herrmann et al. as the reference standard [12]. These thresholds are 7.1, 9.2 and 13 kPa, for significant fibrosis (≥F2), severe fibrosis (≥F3) and cirrhosis (F4), respectively [12]. To be consistent with the aforementioned study, subjects with HBV-related CLD (n = 15) were not included in the analysis as they might have shown slightly lower cut-off points for each fibrosis stage. Consequently, the final analysis to establish specific cut-off values for fibrosis staging included 228 non-HBV subjects with successful measurements for both SSI and X+pSWE.

The AUROCs of X+pSWE for the staging of significant fibrosis (F2), severe fibrosis (F3) and cirrhosis (F4) were 0.96 (95% CI, 0.93–0.99), 0.98 (95% CI, 0.97–1) and 0.99 (95% CI, 0.98–1), respectively. The best cut-off values for diagnosing fibrosis ≥F2, ≥F3 and F4 were, respectively, 6.9, 8.5 and 12 for X+pSWE. Accuracy, sensitivity, specificity, and positive and negative predictive values for each fibrosis threshold are reported in Table 3.

### 3.5. Cut-Off Performance in the “Liver Biopsy” Subgroup

The effectiveness of the aforementioned cut-off values to establish fibrosis staging was evaluated in the subgroup of patients who underwent liver biopsy (n = 120 patients). In this group the majority of patients showed an early stage of fibrosis after histopathology evaluation (85 patients with no or mild fibrosis and 18 patients with moderate fibrosis). As only 17 patients showed an advanced fibrosis (F3) or cirrhosis (F4) these were merged together into a single fibrosis group (F ≥ 3) in the statistical analysis. Technical failures/unreliable LS measurements occurred in seven patients with X+pSWE and two patients with SSI, who were then excluded from this analysis. Overall, SSI correctly classified 99 out of 118 patients (84%) for F ≥2 and 107 out of 118 (91%) patients for F ≥ 3, while X+pSWE identified 93 out of 113 patients (82%) for F ≥ 2 and 101 out of 113 patients (89%) for F ≥ 3. The global performance of both SWE techniques in differentiating significant fibrosis (≥F2) and advanced fibrosis (≥F3) is shown in Table 4.

## 4. Discussion

In the present study we tested, for the first time, the feasibility and the diagnostic accuracy of the X+pSWE technique as compared to another well-validated SWE devices and/or liver histology. Overall, LS measurement with both elastography techniques showed good feasibility in clinical practice. In particular, the feasibility of X+pSWE was comparable to SSI in this cohort of patients (96% and 98%, respectively). These excellent results were achieved despite the high prevalence of obese patients (37%) in our study group, which typically represents a major limitation for both US imaging and liver stiffness measurement. In fact, the main obstacle for both SWE techniques was a skin–liver capsule distance of more than 5 cm, typically encountered in morbid obesity, that in some patients did not allow the placement of the ROI adequately below the liver capsule.

In recent years, due to the decline in performing liver biopsies for “simple” indications such as fibrosis staging, the first step to validate a new SWE device is to compare its diagnostic accuracy against another previously validated SWE technique (such as TE or SSI) [14]. Our results showed that LS measured with X+pSWE and SSI had an excellent correlation (r^2^ = 0.90), with a mean difference between the two techniques showing average values only slightly lower for X+pSWE (−0.24 kPa). The concordance between the systems also holds for values of LS reflecting severe fibrosis/cirrhosis (>10 kPa), which is not always the case when comparing different SWE systems. In this regard, a thorough and elegant study by Ferraioli et al. comparing six different SWE devices with TE showed that variability between measurements occurred especially when evaluating patients with higher degrees of fibrosis/LS (F > 2). On the contrary, concordance was higher when the values obtained by the various machines was <15 kPa [15].

Another important strength of our study is the identification of cut-off values for non-invasive stratification of liver fibrosis stages. Previously published data on the diagnostic accuracy of US devices integrated with pSWE software often showed lower cut-off values compared to TE and 2D-SWE [14,16]. In our cohort the cut-off values of X+pSWE for staging liver fibrosis were only slightly lower than those of SSI across all stages of liver fibrosis, with a maximal difference of 1 kPa to establish a diagnosis of cirrhosis (12 kPa for X+pSWE vs. 13 kPa for SSI). Obviously, an excellent agreement does not always mean that the values are absolutely overlapping but only that there is concordance between them because they follow the same direction. For instance, when employing some SWE techniques, the differences between values obtained with different US devices may reach differences of >2 kPa [15]. Therefore, the threshold values for fibrosis staging cannot always be interchangeably applied across different US systems. In fact, it is known that cut-off values for fibrosis staging may vary across US systems from different vendors, but this variance has somewhat decreased in recent years due to the efforts of the Quantitative Image Biomarker Alliance (QIBA, an organization composed of scientists, clinician, US manufacturers and members of the U.S. Food and Drug Administration) that developed specific phantoms that help to standardize LS measurements [17]. Moreover, from a clinical point of view, rather than providing an exact fibrosis staging using the METAVIR or similar scoring systems, it might be more important in clinical practice to rule in or rule out significant disease. On this basis, the results obtained from our study are in line with the recommendation of a recent consensus of the SRU (Society of Radiologists in Ultrasound), which proposes a simplified and more clinically relevant vendor-neutral “rule of four” for the interpretation of liver stiffness values obtained by all SWE techniques regardless of the specific US commercial device [17]. Nevertheless, the results of this study show that the agreement between measurements of LS performed with the two different US systems is strong and that diagnostic accuracy of X+pSWE is also good when using histology as a reference standard.

This study had limitations. First, the intra- and inter-observer variability was not assessed. We decided this in order to avoid discomfort to the patient by prolonging the complete scanning and also to limit exposure to high frequency US. Besides, the main objective of this study was to assess the variability between the two SWE systems and to establish cut-off values for liver fibrosis staging. Another drawback of the study is the lack of histology reference for the whole study group due to the progressive reduction of clinical demand for liver biopsies especially in patients with virus-related CLD. Nevertheless, we showed that a significant subgroup of patients (n = 120), including those in the “difficult to scan” population such as overweight/obese patients, who underwent the liver biopsy X+pSWE technique demonstrated a good diagnostic accuracy to diagnose moderate fibrosis (F ≥ 2) and severe fibrosis (F ≥ 3). A further partial limitation is the heterogeneous distribution of CLD etiologies, with a prevalence of NAFLD, and fibrosis stages, with a majority of patients in the early phases of disease-course (non-clinically significant fibrosis), which did not allow sophisticated subgroup analysis. Anyhow, the request for liver biopsy for patients with advanced fibrosis is decreasing everywhere due to the effectiveness of non-invasive diagnostic tools in liver cirrhosis. We think that our cohort, showing a high prevalence of NALFD patients (>150 subjects), reflects the “real world” scenario that is usually encountered by hepatologists in daily care. In addition, the patients that we enrolled were a selected cohort referred from a large tertiary care medical center with US operators that are familiar with most of the SWE devices available on the market; thus, the study findings may not reflect results in a more general population with liver elastography performed by ultrasonographers/radiologists not adequately skilled in multi-parametric US imaging.

## 5. Conclusions

In the present study conducted in a large cohort of CLD patients we showed that correlation between the new X+pSWE technique and the validated 2D-SWE SSI in LS measurements is very good and that mean LS values obtained with this pSWE system are in line with those of SSI. Our analysis suggests for the first time cut-off thresholds for fibrosis staging using X+pSWE. Further studies are warranted in order to confirm these proposed cut-off values in larger populations with different CLD etiologies (including HBV patients).

## Figures and Tables

**Table 1:** Clinical and laboratory findings of the patients enrolled in the study.

Patient Demographics (n = 253)	
Age (years)	54 (18–89)
Sex (male)	128 (51%)
BMI (kg/m^2^)	29 ± 7
BMI distribution	77 (30%)
<25 (underweight/normal range)	82 (33%)
25–29.9 (overweight)	94 (37%)
>30 (obese)	
Etiology (all patients)	
NAFLD	153 (60%)
PBC/PSC/AIH	33 (13%)
Hepatitis C virus	19 (8%)
*-Naïve*	4 (21%)
*-Treated*	15 (79%)
Hepatitis B virus	15 (6%)
Other	33 (13%)
Bilirubin (mg/dL)	1 (0.4–5)
Albumin (g/dL)	4.2 (2.5–5.1)
INR	1 (0.89–1.5)
ALT (U/L)	40 (6–290)
AST (U/L)	35 (35–291)
γ-GT (U/L)	66 (9–379)
ALP (U/L)	73 (24–383)
Platelet count (×10^9^/L)	199 (57–482)

**Note:** Results are expressed as median (range), mean (± standard deviation) or number (%); n, number of patients; BMI, body mass index; NAFLD, non-alcoholic fatty liver disease; PBC, primary biliary cholangitis; PSC, primary sclerosing cholangitis; AIH, autoimmune hepatitis; INR, international normalized ratio; ALT, alanine aminotransferase; U/L, units per liter; AST, aspartate aminotransferase; γ-GT, gamma-glutamyl transferase; ALP: alkaline phosphatase.

**Table 2:** Median values (range) of LS obtained with the different SWE devices according to fibrosis stage (SSI as a reference standard).

SWE Device	F0–1	F2	F3	F4
SSI (kPa)	5.1 (3.2–7)	8 (7.1–9.0)	11.1 (9.4–12.8)	18.5 (13.1–33.2)
*n of patients*	*164*	*27*	*30*	*27*
X+pSWE (kPa)	4.9 (2.9–8.9)	7.8 (4.5–9.9)	10.3 (5.9–14.8)	17.3 (11.1–38.1)
*n of patients*	*162*	*26*	*28*	*27*

**Note:** SWE, shear wave elastography; SSI, 2D-SWE Supersonic Imagine; kPa, kilopascal; X+pSWE, X+ point-SWE Alpinion; n, number; F, fibrosis stage.

**Table 3:** Diagnostic accuracy of X+pSWE technique (SSI as a reference standard).

Parameter	F ≥ 2	F ≥ 3	F = 4
Cut-off (kPa)	6.9	8.5	12
AUROC	0.96	0.98	0.99
Sensitivity (%)	92	92	92
Specificity (%)	93	96	98
PPV (%)	85	86	89
NPV (%)	96	97	99
Accuracy (%)	93	95	98

Note: X+pSWE, X+ point-SWE Alpinion; SSI, 2D-SWE Supersonic Imagine; F, fibrosis stage; kPa, kilopascal; AUROC, area under the ROC curve; PPV, positive predictive value; NPV, negative predictive value.

**Table 4:** Diagnostic accuracy of SWE techniques (liver histology as a reference standard).

Parameter	SWE Device	F ≥ 2	F ≥ 3
Cut-off (kPa)	SSI	7.1	9.2
	X+pSWE	6.9	8.5
AUROC	SSI	0.90	0.96
	X+pSWE	0.89	0.90
Sensitivity (%)	SSI	79	88
	X+pSWE	75	87
Specificity (%)	SSI	86	91
	X+pSWE	85	90
PPV (%)	SSI	69	61
	X+pSWE	67	57
NPV (%)	SSI	91	98
	X+pSWE	90	98
Accuracy (%)	SSI	84	91
	X+pSWE	82	89

**Note:** SWE, shear wave elastography; F, fibrosis stage; kPa, kilopascal; SSI, 2D-SWE Supersonic Imagine; X+pSWE, X+ point-SWE Alpinion; AUROC, area under the ROC curve; PPV, positive predictive value; NPV, negative predictive value.

## Data Availability

The datasets analyzed during the current study are available from the corresponding author on reasonable request.
